# Vitamin D: Possible Therapeutic Roles in Hepatocellular Carcinoma

**DOI:** 10.3389/fonc.2021.642653

**Published:** 2021-05-25

**Authors:** Isaacson B. Adelani, Oluwakemi A. Rotimi, Emmanuel N. Maduagwu, Solomon O. Rotimi

**Affiliations:** ^1^ Department of Biochemistry, Covenant University, Ota, Nigeria; ^2^ Department of Biochemistry, Chrisland University, Abeokuta, Nigeria

**Keywords:** vitamin D, therapeutic, inflammation, apoptosis, differentiation, proliferation, hepatocellular carcinoma

## Abstract

Hepatocellular carcinoma (HCC) is a unique type of liver cancer instigated by underlying liver diseases. Pre-clinical evidence suggests that HCC progression, like other cancers, could be aided by vitamin D deficiency. Vitamin D is a lipid-soluble hormone usually obtained through sunlight. Vitamin D elucidates its biological responses by binding the vitamin D receptor; thus, promoting skeletal mineralization, and maintain calcium homeostasis. Other reported Vitamin D functions include specific roles in proliferation, angiogenesis, apoptosis, inflammation, and cell differentiation. This review highlighted studies on vitamin D’s functional roles in HCC and discussed the specific therapeutic targets from various *in vivo*, *in vitro* and clinical studies over the years. Furthermore, it described recent advancements in vitamin D’s anticancer effects and its metabolizing enzymes’ roles in HCC development. In summary, the review elucidated specific vitamin D-associated target genes that play critical functions in the inhibition of tumorigenesis through inflammation, oxidative stress, invasion, and apoptosis in HCC progression.

## Introduction

Hepatocellular carcinoma (HCC) is a unique type of liver cancer instigated by underlying liver diseases. In general, liver cancer constitutes a substantial public health problem that ranks as the sixth most commonly diagnosed cancer and the third most common cause of cancer-related mortality in 2020 (1). Although liver cancer occurs in both genders, the incidence and mortality rates in males are 2 to 3 times higher than in females (1). The loss of the liver’s regenerative ability exacerbates HCC progression, which subsequently potentiates organ failure (2). This loss of regenerative capacity is further compounded by the disruption of various pathways associated with the pathogenesis and progression of HCC, thereby making HCC an outcome of a complex cascade of events (3). Furthermore, the increasing incidence of HCC is mainly associated with viral infections, including hepatitis B (HBV) and C viruses (HCV), as well as other risk factors like non-alcoholic fatty liver disease (4, 5) and mycotoxin exposure (6–9). Aside from these biotic, lifestyle, and environmental factors, pre-clinical evidence suggests that HCC progression, like many other cancers, could be facilitated by vitamin D (VD) deficiency and germline genetic variants in the Vitamin D receptor (*VDR*) gene, which has been shown to influence the progression of hepatitis to HCC (10, 11). Also, an epidemiological study showed that increased maternal ultraviolet (UV) exposure is associated with a reduced risk of hepatoblastoma in offspring (12). Additionally, evidence from the SEER data showed that the incidence of HCC in the United States is associated with ambient UV exposure (13). Hence, this premise supports the VD - cancer hypothesis and further augments the roles of vitamin D metabolism in hepatocellular carcinogenesis (2).

Vitamin D (VD) is a lipid-soluble hormone usually obtained through the exposure of skin to sunlight. Several factors, including skin pigmentation and a modern lifestyle, could limit VD formation, thus causing VD deficiency (14). In VD synthesis, sunlight UV (B) (280–315 nm) exposure on the skin activates 7-dehydrocholesterol to pre-vitamin D_3_ and eventually cholecalciferol (VD_3_) (2). Similarly, UV (B) exposure to ergosterol in plants and fungi produces another form of vitamin D, ergocalciferol (VD_2_) (15). Asides from the endogenous synthesis of VD, VD_3_ can also be obtained from diets while VD_2_ is principally used during vitamin D fortification. Both forms of VD are naturally inactive and are activated *via* hydroxylation. After synthesis, VD binding protein (*DBP*) binds VD and transports it to the liver, where hydroxylation at carbon-25 metabolizes VD to 25-hydroxyvitamin D (25(OH)D) through *25-hydroxylase*. In this first phase of VD metabolism, hydroxylation occurs predominantly in the hepatic cells although extrahepatic VD hydroxylation reportedly occurs in other tissues with evident 25-hydroxylase activities (16, 17). Most importantly, during this first hydroxylation step, an ubiquitous mitochondrial 25-hydroxylase, CYP27A1 does not hydroxylate VD_2_ whereas, CYP2R1 usually located in the liver and testes hydroxylates both forms of VD (15). Equally, Zhu et al. (18) reported CYP2R1 as a major but not the only 25-hydroxylase. After the first hydroxylation, the glomerulus filters 25(OH)D transported into the kidney and converts it to a steroid hormone (the active form of VD), 1α, 25 (OH)_2_D (calcitriol), through *25(OH)D-1α-hydroxylase* (19). This metabolic activity in the kidney signifies the second stage of VD hydroxylation. Although 1α-hydroxylation occurs predominantly in the kidney, peripheral tissues including the skin and lymph nodes exhibit extra-renal production of the steroid hormone (20). Finally, in a bid to activate VD’s biological response to regulate gene expression, calcitriol binds *VDR* (17) in a binding sequence that allows the effective functioning of retinoid X (*RXR*). *RXR* belongs to the nuclear receptor family and a member of the steroid/thyroid hormone, primarily functioning as transcription factors (21). *RXR* also plays essential roles in metabolism and cell differentiation (21). Hence, VD binding enables the *VDR – RXR* interaction, leading to VD-related functions through gene transcription (22). Thus, VD’s biological action is dependent on *VDR*, *RXR*, and the availability of VD (23).

Asides from primary functions, which include promoting skeletal mineralization and maintenance of calcium homeostasis, VD performs pro-apoptotic, pro-differentiation, anti-angiogenetic, anti-proliferative, anti-invasive, and anti-metastatic functions (24). Reports show that VD is an indicator of HCC prognosis and could be vital in predicting HCC patients’ mortality (25). Meanwhile, VD deficiency is fast becoming a global public health challenge (26), and it is continuously associated with an ‘all-cause and cause-specific mortality, despite differences in the VD baseline levels across the world (27, 28). Consequently, there are pieces of evidence showing connections between VD deficiency and HCC progression. For instance, Gaksch et al. (29) meta-analysis proposed an inverse relationship between serum VD (25(OH)D_3_) level and HCC risk; thereby, suggesting VD’s prospective therapeutic ability in managing HCC. Moreover, increased bioavailability of circulating 25(OH)D_3_ was also associated with HCC survival as against total or free VD level (30). In contrast, Liu et al. (31) reported that increased 25(OH)D level was associated with an increased risk of HCC incidence. However, they observed that genetic variations related to VD metabolism could influence HCC tumor response, survival, and mortality.

Despite the reported association with HCC development, contrasting reports suggest that baseline VD level could play little or no role in cirrhosis-linked HCC (32). Therefore, this review highlights the *in vivo*, *in vitro*, and clinical studies on VD therapeutic targets in HCC. Furthermore, it discussed the significant limitations and possible solutions in using VD as therapeutics.

## VD, VDR, and HCC Pathological Conditions

The progression of HCC and pathological conditions like liver cirrhosis are linked to VD deficiency; hence, suggesting that decreased 25(OH)D is associated with poor liver disease prognosis (33). According to Berkan-Kawińska et al. (34) and Yang et al. (35), patients with liver cirrhosis, HBV, and HCV have decreased 25(OH)D levels and could benefit from VD supplementation. VD deficiency has also been linked with infections in patients with HCV-associated liver cirrhosis (36) and the VD deficiency-associated polymorphisms, like rs1993116, rs10741657, rs2282679, rs7944926, and rs12785878, linked with HCV-related HCC (37). This study by Lange et al. (37) also showed that reduced 25(OH)D_3_ levels in HCV-related HCC patients is associated with genetic variations of CYP2R1, GC, and DHCR7. While the circulating form of VD (25(OH)D_3_) instigates the hormone’s anti-HCV capacity (38), the active form of VD (1α, 25 (OH)_2_D_3_) induces *CYP24A1* expression in a *VDR*-dependent manner. However, *VDR* expression, repressed by HBV transcript upregulation, affects VD’s binding to the receptor (39). Also, chronic HBV patients are at a higher risk of increased VD deficiency (40).

HCV and HCV-related HCC patients had lower levels of VD and VDR compared to healthy individuals (41). In the same vein, Falleti et al. (42) reported that VDR polymorphisms are associated with the occurrence of HCC in liver cirrhosis patients, specifically in those with alcoholic etiology. In the study, HCC was linked with the b allele of the BsmI A>G (B/b) polymorphism and the T allele of the TaqI T>C (T/t) polymorphism (42). Several studies have reported relationships between VDR polymorphisms and HCC pathological conditions. In a Chinese population hospital-based case-control study, VDR rs2228570 and DBP rs7041 polymorphisms vary between HBV-related HCC patients and healthy individuals thus, suggesting a relationship with increased risk of HBV-related HCC (11). A meta-analysis strengthened these observations, which indicated that VDR rs7975232 and rs2228570 polymorphisms are associated with HCC (43).

Although a non-association of VDR polymorphism and risk of HBV infection in Vietnamese HBV patients was reported by Hoan et al. (44), they suggested that Apal VDR polymorphism (rs7975232) could be associated with clinical outcomes and disease progression. Incidentally, Apa1 VDR polymorphism was shown to be associated with HCC in HCV-cirrhotic patients (45). On the contrary, SNPs of VDR at BsmI, ApaI, and TaqI loci showed no difference between HCC and non-HCC patients, according to Yao et al. (46). However, the authors reported a higher frequency of VDR FokI C > T polymorphism in HCC patients. Also, HCC patients showed a higher prevalence of FokI TT genotype, which is a risk factor for HCC development (46). Interestingly, the FokI TT genotype was also associated with HCC clinicopathology characterized by increased serum alpha-fetoprotein (AFP), advanced tumor stage, cirrhosis, and lymph node metastasis (47). Besides, the Fok1 T allele is linked with a predisposition to reduced VD levels and an increased probability of cancer development in HCV patients (47).

Since there are associations between VD, VDR, and HCC pathological conditions, understanding VD-related mechanisms and therapeutic targets in HCC progression could further substantiate existing evidence and highlight the roles of the hormones in hepatocarcinogenesis.

## Therapeutic Effects of Vitamin D in HCC

### *In Vitro* Studies

Over the years, there have been reports of HCC’s resistance to many drugs. An example is resistance to Everolimus, which acts as an *mTOR* (mechanistic target of rapamycin) inhibitor. *mTOR* is a serine/threonine-protein kinase found in the PI3K-related kinase (PIKK) family. *mTOR’s* activation plays critical roles in cell metabolism, proliferation, and HCC progression (48). Hence, inhibiting *mTOR* is one of the suggested therapeutic targets used to prevent and manage HCC (49). A recent study reported that calcitriol treatment could restore HCC cell sensitivity, thus becoming less resistant to everolimus (50). The reduced cell resistance modulated through the epithelial-mesenchymal transition pathway increased expression of miRNA-375 and decreased expression of target genes, including Metadherin (*MTDH*), Yes-associated protein-1 (*YAP-1*), and cellular Myc (*c-MYC*) (50).

Likewise, Huang et al. (51) investigated calcitriol’s effects on Histone deacetylase 2 (*HDAC2*) and cell cycle markers to explore the senescence and apoptotic pathway involved in HCC. According to Huang et al. (51), silencing the *HDAC2* gene, which is usually highly expressed in HCC tumors, enhances calcitriol’s inhibitory effects. Equally, 1,25(OH)_2_D_3_ treatment decreased the expression of *HDAC2* with a dose-dependent increased expression of cell cycle marker, cyclin-dependent kinase inhibitor *(p21(WAF1/Cip1))* (51). This result suggests that VD could be a potential therapeutic agent in managing HCC *via* cell cycle modulation. However, VD_3_ treatment significantly increased Thioredoxin Interacting Protein (*TXNIP*); thus, enhancing apoptosis while reducing cell proliferation and thioredoxin activities (52). *TXNIP* is a tumor suppressor gene usually downregulated in HCC; therefore, instigating HCC progression (53). Furthermore, in its hormonal form, VD (1,25(OH)_2_D_3_) exhibits anti-proliferative ability and increases the apoptotic ratio in HCC cell lines (54). 1000 nM VD treatment also showed potential cell growth ameliorating ability in HCC cell lines according to the study of Xu et al. (51). Although VD reduced cell viability and proliferation while activating apoptosis, the effects were well enhanced when co-administered with Astemizole (a non-sedating antihistamine). In the same study, VD’s anti-invasive, anti-tumor, and cell migration inhibitory properties were highlighted (55).

Recently, a combination of VD_2_ analog, Doxercalciferol, and Carnosic acid-enhanced Sorafenib induced HCC cell death through blockage of autophagosomes/lysosomes fusion while also activating autophagy and apoptosis (56). To further elucidate the more apparent HCC related mechanisms, Wang et al. (57) showed that 1,25(OH)_2_D_3_ reversed biological alterations of hepatic progenitor cells caused by Aflatoxin B1 (AFB1) in WB-344 cells. Furthermore, VD_3_ attenuated the activation of Protein kinase B *(Akt)* while suppressing the expression of cysteine-rich angiogenic inducer 61 *(CYR61)* and connective tissue growth factor *(CTGF)*, thus indicating anti-tumor effects. Calcitriol, also showed inhibitory roles in HCC by suppressing the hepatocyte growth factor (*HGF*) and its receptor, *c-met* (58).

Therefore, it can be deduced from these *in vitro* studies as summarized in **Table 1** that VD acts as an anti-tumor agent in HCC, and it could regulate tumor growth/progression through cell cycle modulation and mTOR inhibition.

**Table 1:** Summary of the effects of vitamin D on HCC targets genes.

Effects of vitamin D on *in vitro* HCC targets					
S/N	Vitamin D dosage (duration)	HCC cell lines	Target genes (method)	Summarized findings on vitamin D effects	References
**1.**	1, 10, 100 or 1000 nM (48 hours)	H22 and Hepa1–6	NA (Colony formation, Annexin V and PI double-staining)	1,25(OH)_2_D_3_ reduced cell proliferation and induced apoptosis.	(54)
**2.**	0, 10, 100 or 500 nM (24, 48, 72hrs)	Huh7, HepG2, and Hep3B	*TXN*	VD_3_ had no significant effect on TXN and CDNK1B	(52)
			*CDNK1B*		
			*CDNK1A*	VD_3_ downregulated the expression of CDNK1A.	
			*TXNIP*	VD_3_ upregulated the expression of TXNIP.	
**3.**	10−7 M (12/24 hrs pre-treatment; 21 days co-treatment with Everolimus)	PLC/PRF/5 EveR and JHH-6 EveR	NA (Colony formation and cell proliferation)	1α, 25 (OH)_2_D restored everolimus sensitivity to everolimus-resistant (EveR) HCC cell lines	(50)
	10−7 M for 6 days		E-cadherin, cytokeratin 18, and vimentin (WB and IF)	1α, 25 (OH)_2_D caused EMT induction through decreased expression of vimentin and increased expression of E-cadherin and cytokeratin-18.	
	12hrs and 6 days of treatment.		*MTDH, YAP-1*, and *c-MYC* (WB)	While 12hrs of 1α, 25 (OH)_2_D treatment upregulated miR-375 expression, 6 days of treatment reduced expression of miR-375 targets *MTDH, YAP-1*, and *c-MYC.*	
**4.**	0, 0.1, 1, 10, 100 or 1000 nM	HpG2	*HDAC2, p21(WAF1/Cip1)* (Reverse transcription, WB)	1,25(OH)_2_D_3_ caused a dose-dependent decrease in the HCC growth rate. 1,25(OH)_2_D_3_ also decreased the mRNA expression and protein level of *HDAC2* and increased the expression/protein level of *p21(WAF1/Cip1).*	(51)
**5.**	1.0, 10.0 nM (5hrs)	HepG2, Huh-Neo, Huh5-15, and Hep3B	*CYP24A1, CYP27B1*, and *VDR* (qRT-PCR, IHC)	1,25(OH)_2_D_3_ increased the expression of *CYP24A1*	(59)
**6.**	0.1, 1, 10, 100 or 1000 nM (24 hours)	HepG2 and SMMC-7221	NA (Cell viability and proliferation)	Astemizole (1–2 μM) increased VD-induced (>100 nM) cell viability and proliferation reduction, cell invasion, increased pro-apoptotic effects, and upregulated VDR expression-induced anti-tumorigenic effects.	(55)
**7.**	100nM (14 days)	WB-F344	*CD133, EpCAM, HNF4α, CK19* (FC, WB, Cell viability)	1,25(OH)_2_D_3_ inhibited colony formation, cell viability of WB-334 and promoted apoptosis.	(57)
			*Cyclin D1, p27, lats1, YAP, TAZ*, *CYR61, CTGF* (WB)	1,25(OH)_2_D_3_ caused a partial reversal of *AKT* phosphorylation (at Ser473) and gene alterations of *cyclin D* and *p27kip*. 1,25(OH)_2_D_3_ blocked *YAP/TAZ* activation and *LATS1* dephosphorylation.	
**8.**	0.01–1 μ M (7 days)	HepG2 and Hep3B		VD inhibited cell proliferation. VD also altered cadherin/catenin adhesion through an increased level of β-catenin in Smad3^+/−^ MEF cells as well as knockdown of Smad3 and *VDR* in HepG2 cells	(60)
**9.**	100 nM Doxercalciferol	Huh7 and HCO2	BIM, Cas 9, Cas 3, Beclin1, Atg3, LC3-II	The combination of Doxercalciferol, Carnosic acid, and sorafenib increases the expression of apoptosis and autophagy-related proteins.	(56)
**Effects of vitamin D on *in vivo* HCC targets**					
**S/N**	**Vitamin D dosage (duration)**	**Host organism**	**Target genes (method)**	**Summarized findings on vitamin D effects**	**References**
**10.**	0.1 μg/kg (14 days)	Mice (HCC through orthotopic transplantation)	*IL-6, TNF-α* (ELISA)	Exogenous supplementation of VD reduced inflammatory cytokines in 1α(OH)ase knockout mice.	(54)
**11.**	0.3 µg/100µl (4-20 weeks)	Rats		VD_3_ induced antioxidant defense system	(61)
**12.**	N/A	Human	*VDR, VDUP-1* (Reverse transcription)	25(OH)D was reduced in HCC patients with concomitant increased *VDR*, and *VDUP-1* mRNA upregulated expression.	(62)
**13.**	200 IU/kg (daily for 16 weeks)	Rats	*Nrf2, TGF-β1, Cas-3* (Reverse transcription, ELISA)	VD_3_ triggered hepatoprotective effects while enhancing the anti-tumor effects of 5-fluorouracil. It regulates cancer progression through downregulation of *Nrf2, TGF-β1* and induces apoptosis by upregulating *Cas-3*.	(63)
**14.**		Pig	NA	Administration *via* hepatic artery rather than intravenous route could allow for an increased dosage of VD	(64)
**15.**	100nM	Mice	*CK19* (IHC)	1,25(OH)_2_D_3_ protected the liver integrity by reducing serum ALT, AST, and *CK19* cells initially increased with AFB-1 administration.	(57)
**16.**	200 IU/kg and 10000 IU/kg (4 months)	Mice	*PDCD4, p21, p27, p53, Akt, c-Myc, mTor, Stat5A, Bcl-XL, PEA15, cyclin D1*	Repression of tumor suppressors and induction of oncogenic proteins are associated with VD deficiency.	(60)
**Effects of vitamin D in clinical trials**					
**17.**	50, 75, 100 µg (4 weeks)	Human	NA	Co-administration with lipiodol could increase a safe dosage without hypercalcemia complications. Also, the co-administration stabilized HCC patients through the mediation of tumor marker, AFP.	(65)
**18.**	5 - 20 µg/day seocalcitol	Human	NA	Complete responses in some patients after Seocalcitol treatment showed that the analog could help stabilize HCC patients and may possess the anti-tumorigenic ability.	(66)
**19.**	50000 IU weekly (26 weeks)	Human	*TBR1, TBR2, Smad3, Smad4, and β 2SP* (IHC)	VD treatment repressed β –catenin expression while inducing the expressions of TBR2, Smad3 in HCC patients. The study showed that VD treatment could restore TGF-β signaling in cirrhosis and liver cancer patients.	(60)
**20.**	2800 IU daily (8 weeks)	Human		In cirrhotic randomized control trial patients, VD_3_ supplementation significantly increased 25(OH)D serum concentrations. However, the supplementation with VD_3_ had no significant effect on liver function, fibrotic and mineral metabolism parameters.	(67)

EMT, epithelial-mesenchymal transition; TXN, thioredoxin; TXNIP, thioredoxin interacting protein; HNF4α, hepatocyte nuclear factor 4 alpha; CDNK1, cyclin-dependent kinase inhibitor 1; YAP, Yes-associated protein; TAZ, transcriptional co-activator with PDZ-binding motif; HDAC2, histone deacetylase 2; MTDH, metadherin; EpCAM, epithelial cell adhesion molecule; CYP24A1, cytochrome P450 family 24 subfamilies A member 1; CYP27B1, cytochrome P450 family 27 subfamily B member 1; CYR61, cysteine-rich angiogenic inducer 61; CTGF, connective tissue growth factor; FC, flow cytometry; WB, Western blotting; qRT-PCR, quantitative reverse transcription PCR; AFB-1, aflatoxin B-1; Nrf2; TGF-β1, tumor growth factor B-1; IL-6, interleukin 6; TNF-α, tumor necrosis factor-alpha; Cas-3, caspase 3; VDR, vitamin D receptor; VDUP-1, vitamin D3-upregulated protein-1; CK19, cytokeratin 19; IHC, immunohistochemistry.

### *In Vivo* Studies

**Figure 1:**
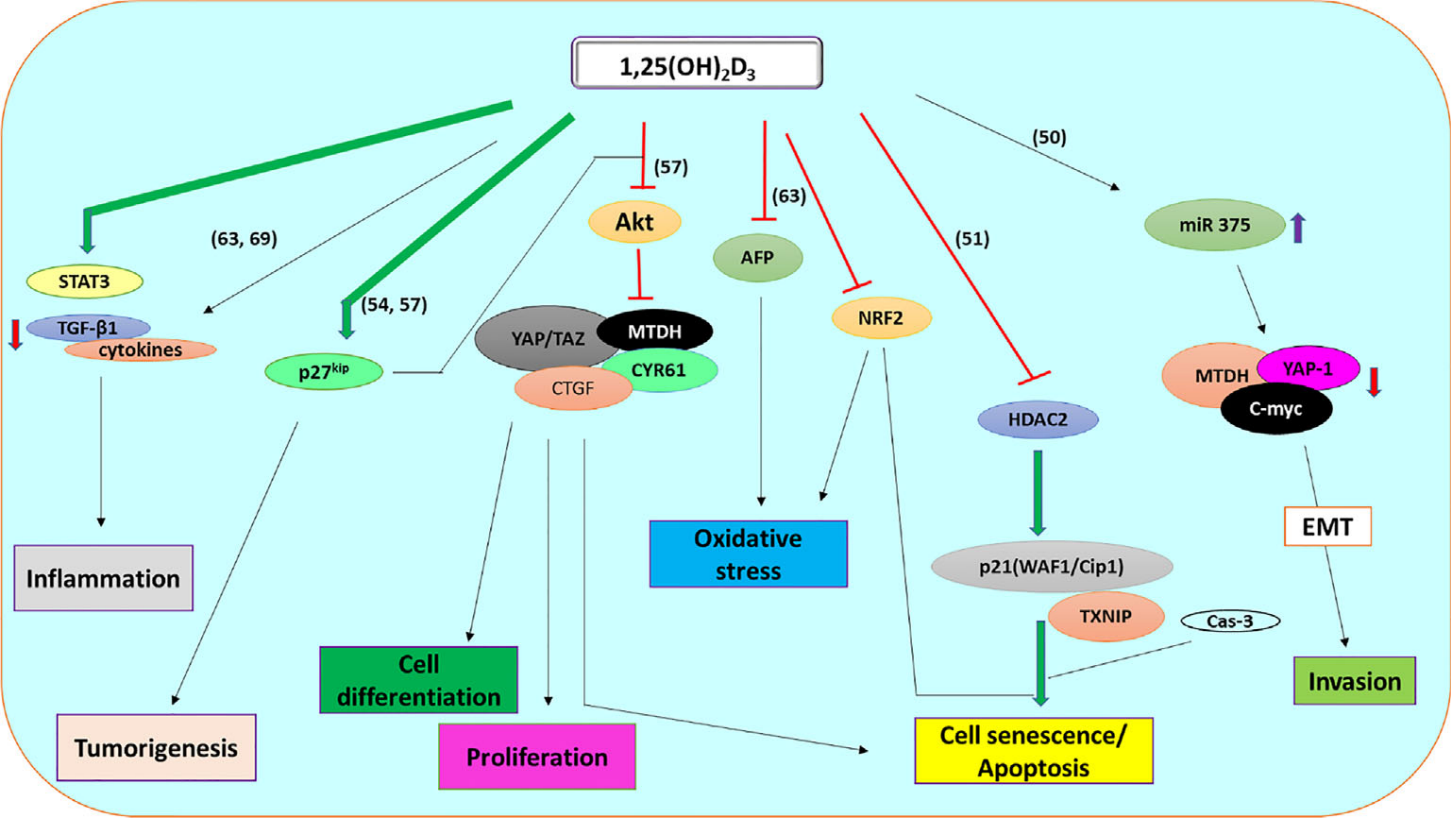
l,25 (OH)_2_D_3_ signaling pathway involved in the regulation of HCC through apoptosis, invasion, proliferation, differentiation, tumorigenesis, oxidative stress, and inflammation.

VD’s anti-inflammatory role in carcinogenesis is now considered an established mechanism of its anti-carcinogenesis property (68). For example, in an activated inflammatory response, dietary VD significantly ameliorated cytokine production observed with diethylnitrosamine (DEN) effects in rats (69). Similarly, a deficient state of 1,25(OH)_2_D_3_ triggers inflammatory cytokines production through *STAT3* activation (**Figure 1**) (50). Guo et al. (54) also linked the anti-tumor ability of 1,25(OH)_2_D_3_ with the availability of *p27^kip1^* in mice (54). *p27^kip1^* is a cyclin-dependent kinase inhibitor known for its prognostic roles in carcinogenesis. Asides from functioning as a tumor suppressor, *p27^kip1^* promotes apoptosis, regulates tumor drug resistance, protects against inflammatory effects, and enhance cell differentiation as summarized in **Table 1** (70). Also, the loss of *p27^kip1^* could negatively affect the anti-tumor ability of 1,25(OH)_2_D_3_. The ablation of kidney VD metabolic enzyme, 25(OH)D_3_-1α-hydroxylase, resulted in tumor formation and increased inflammatory responses in mice (54). However, in the DEN-induced hepatocarcinogenesis mice model, loss of VD_3_ upregulated protein 1 (*VDUP1*) promotes carcinogenesis through increased cell proliferation, expression of tumor necrosis factor-α (*TNF-α*) and nuclear factor-kappa B *(NF-kB)* activation, thus suggesting VDUP1 as a potential anti-proliferative therapy target (71).

Generally, inflammation induces oxidative stress by activating neutrophils and Kupffer cells, which subsequently triggers cancer progression (72). Oxidative stress is usually associated with the pathogenesis and progression of HCC. However, reports suggest that VD_3_ could be involved in the attenuation of oxidative stress (61, 69). The physiological advantage of this abuts the vital role of inhibiting oxidative stress in managing hepatocarcinogenesis (73, 74). Besides, VD_3_ protected against oxidative stress-induced carcinogenesis by reversing different antioxidant enzymes altered in 3’ methyl-4-dimethyl-amino-azobenzene-induced hepatocarcinogenesis (61).

Furthermore, increased serum level and gene expression of the *M30* apoptotic marker in HCC patients, amongst others, indicates alteration of the apoptotic pathway in carcinogenesis (62). Thus, the co-regulatory interaction between VD signaling and apoptotic pathway in HCC is imperative in the understanding of VD-related mechanisms (62). Besides, VD_3_ (cholecalciferol) treatment activates caspase 3 (*Cas-3*) expression while downregulating protein expression of tumor growth factor (*TGF-β*) (63). Likewise, co-administration with 5-fluorouracil alleviated the increased liver function enzymes, alpha-fetoprotein (AFP), and nuclear factor erythroid 2-related factor 2 (*Nrf2*) expression in thioacetamide-induced HCC (63).

Therefore, the *in vivo* studies showed that VD could regulate HCC progression *via* activation of apoptosis, reducing oxidative stress and inflammatory effects (**Table 1**).

### Clinical Studies

Despite promising data from *in vitro* and *in vivo* studies suggesting VD’s crucial roles in carcinogenesis, established reports and data from clinical studies are still few and far between. These clinical trials included a phase 1 pilot study on VD administration’s effects on serum calcium, hepatic and renal functions by Finlay et al. (75). In the study, HCC patients received up to a 20-fold increased l,25-(OH)_2_D_3_ *via* hepatic arterial infusion without hypercalcemic complications. The study also reported 10 µg/day as a safe dosage with no renal or hepatic complications (75).

However, to eliminate hypercalcemia effects of VD administration, Morris et al. (74) reported in a relatively small pilot study that co-administration of l,25-(OH)_2_D_3_ with lipiodol in HCC patients could be an excellent therapeutic measure through stabilization of tumor marker, AFP. From this clinical research, the authors suggested that the use of lipiodol could increase permitted l,25-(OH)_2_D_3_ dosage about 50 folds (100 µg) without complications of hypercalcemia. Hence, this positive outcome could have resulted from the intra-arterial hepatic administration route used in the study (65). In addition, Dalhoff et al. (66) administered a starting dose of 10 µg/day seocalcitol (VD analog) and reported that seocalcitol could function as an anti-tumorigenic agent in phase 2 clinical trials. The analog can thus stabilize HCC patients due to its cytostatic rather than cytotoxic capacity (66).

VD may also improve HCC by restoring initially lost tumor growth factor-β (*TGF-β*) expression in liver tumor (60). In support of this, Chen et al. (63) reported that dysregulated VD-associated genes, including Foxhead box protein O4 (*FOXO4*) and signal transducer and activator of transcription 1 (*STAT1*), showed a strong correlation with TGF-β, while VD supplementation reduces cell proliferation.

Furthermore, a selected European population Nested Case-Control Study reported that increased concentration of hormonal VD, l,25-(OH)_2_D_3_ decreased the risk of HCC (76). This study informed the idea that l,25-(OH)_2_D_3_ treatment could ameliorate HCC development. Likewise, a randomized controlled trial also showed that l,25-(OH)_2_D_3_ supplementation of daily 2800 IU resulted in increased serum l,25-(OH)_2_D_3_ concentration in cirrhotic patients without significantly altering the mineral metabolism parameters (74).

## The Role of Vitamin D Metabolizing Enzymes

Beyond the modulating roles of circulating VD hitherto described, evidence is emerging that these effects are elicited through its metabolizing genes. In this vein, Horvath et al. (59) reported that 1,25(OH)_2_D_3_ treatment caused a concurrent dose-dependent mRNA increased expression of *CYP24A1* at specific time points in some HCC cell lines. The upregulated expression of *CYP24A1* through 1,25(OH)_2_D_3_ treatment suggests a positive correlation between the enzyme and VD serum concentration. Chiang et al. (77) also reported that 1,25(OH)_2_D_3_ cell line treatment induces upregulation of *CYP24A1* expression. Even though 25(OH)D-1α-hydroxylase, *CYP27B1* further augmented the upregulation of CYP24A1, as reported by Bikle et al. (78), its transfection also induced cell arrest at the G0/G1 phase through *p21/p27;* thus, inhibiting tumor cell growth (76). Additionally, single nucleotide polymorphisms of *CYP24A1* are associated with an increased risk of HCV infection in some high-risk Chinese population (79). Specifically, rs6013897 (T>A) was significantly associated with an increased risk of HCV infection. In contrast, rs6068816 (C>T), rs3787557 (T>C), rs6022999 (A>G), and rs2248359 (C>T) were associated with increased risk of chronic HCV infection. Consequently, combining VD_3_ treatment and *CYP24A1* inhibitors could annihilate the increased cytoplasmic expression of *CYP24A1*.

## Limitations of the Use of Vitamin D as Therapeutics

VD intoxication, usually characterized by hypercalcemia, is a significant limitation to the therapeutic use of the hormone in alleviating pathological conditions. Consequently, VD analogs have been used in recent years to reduce hypercalcemic effects. For example, a catabolic metabolite of the prodrug, 27 hydroxy BCI-210 (27-OH BCI-210), was reported to inhibit cancer cell growth (80). Although patients take various supplements, including vitamins, to maintain and improve health and prevent disease occurrences (81), there was no observed association between these supplements and HCC patients’ survival (81).

The daily intake of 100,000 IU or more could cause VD toxicity (68), while an increased intake of up to 2000 fold against the prescribed dosage could lead to renal failure (82). It has also been reported that an annual treatment of 500,000 IU VD_3_ increases fracture risk (83, 84). However, a short-term effect of an accidental overdose of VD_3_ was minimal; the long-term effect could be detrimental, as van den Ouweland et al. (83) reported. In the study, a single overdose treatment of 2,000,000 IU VD_3_ caused no short-term clinical toxicity. Therefore, terminating VD and reduced the consumption of calcium and phosphorus helps in managing hypercalcemia. Other interventions integral to controlling hypercalcemia include glucocorticoids, intravenous hydration, diuretics, and calcitonin (85, 86). Equally, to reduce the VD dosage and improve efficacy, combination therapy of VD and its analogs with other chemotherapeutic agents could be explored.

## Future Perspectives

It is important to note that VD as an anticancer therapeutic agent could be associated with the administration route. Aside from the hepatic arterial infusion of this lipophilic vitamin, intravenous administration could determine, to some extent, the therapeutic effects and rate of its effectiveness (64). Also, VD supplementation and CYP27B1 gene transfection therapy are other plausible options of exploration in managing and treating HCC (77). Although dosage limitation exists, it will be beneficial to understand the interaction of the VD signaling pathway and carcinogenesis at the genetic level. The genetic interactions could focus on specific targets; thus, alleviating risks that arise with the limitation.

Another line of thought in VD’s therapeutic use could involve understanding the mechanisms in VD’s modulatory roles of the tumor microenvironment (TME). Tumor growth, invasion, and metastasis are generally affected by the interactions between the tumors and their respective microenvironments (87). Understanding these bidirectional interactions between the tumor cells and the environment could open up therapeutic targets and regimes in liver cancer treatment (88–90). Although VD influences angiogenesis, metastasis, and cancer progression in TME, the active form of VD, 1, 25 (OH)_2_D_3_ modulates a couple of stroma cells explicitly, suppresses tumor growth, and act as an anti-inflammatory agent within the TME, leading to cancer reduction (91).

## Conclusion

Despite positive research findings on VD’s roles in HCC, resulting limitations hinder its progress as a viable therapeutic agent. Although there might be conflicting reports supporting the roles of serum l,25-(OH)_2_D_3_ in HCC, there are ample *in vitro*, *in vivo* data and some randomized clinical control trials suggesting VD-related mechanism is vital in HCC progression. This research gap could be vital in understanding the mechanisms involved in the VD regulation of HCC. Clinical trials on various combination therapies will also help resolve the research deficiencies recorded in standardizing VD dosage. Therefore, it is strongly recommended that more studies should be carried out on combination therapies of various VD analogs and standard therapeutic agents by targeting crucial genes and pathways involved in VD’s non-classical functions.

## Author Contributions

All authors contributed, read, and agreed to the publication of this manuscript. Conceptualization: IA. Supervision: OR, EM, and SR. Roles/writing—original draft: IA. Writing—review and editing: IA, OR, EM, and SR. All authors contributed to the article and approved the submitted version.

## Conflict of Interest

The authors declare that the research was conducted in the absence of any commercial or financial relationships that could be construed as a potential conflict of interest.
